# Construction and improvement strategies of an age-friendly evaluation system for public spaces in affordable housing communities: a case study of Shenzhen

**DOI:** 10.3389/fpubh.2024.1399852

**Published:** 2024-07-18

**Authors:** Jiwen Han, Hang Ma, Mohan Wang, Jinqi Li

**Affiliations:** ^1^School of Architecture, Southeast University, Nanjing, China; ^2^School of Architecture, Harbin Institute of Technology, Shenzhen, Shenzhen, China

**Keywords:** age-friendly community, affordable housing communities, evaluation system of age-friendly, public space, improvement strategies

## Abstract

Characterized by early construction periods, as the concentration of low-income populations and a high level of aging, affordable housing communities face prominent challenges such as incongruence between age-friendly construction and the needs of the older adult population. It is urgent to provide pathways and tools for identifying age-friendly issues and optimizing the built environment. The systematic evaluation of age-friendly communities serves as the foundation for implementing intervention measures by developers. Therefore, the construction of a scientifically systematic evaluation system becomes an objective necessity for age-friendly community development. Building upon existing research, this study systematically outlines the subjects, processes, methods, and content involved in constructing an age-friendly community evaluation system. By the methods such as factor analysis and analytical hierarchy process (AHP), the study focuses on the public spaces of affordable housing communities in Shenzhen as a case for constructing an age-friendly evaluation system. The empirical validation of the indicator system is conducted, and the application results are resulted into concrete improvement recommendations and action items, aiming to provide a practical, quantitative tool for community age-friendliness evaluation. The study reveals that adhering to an effective evaluation process, exploring collaborations among multiple stakeholders, determining hierarchical evaluation criteria, and adopting diversified evaluation methods are key to constructing an age-friendly evaluation system for communities. Additionally, the specificity of the evaluation system is influenced by regional demographic structures, policy backgrounds, and the built environment.

## 1 Introduction

Due to declining birth rates and improvements in healthcare, global aging has become an irreversible trend, with the average life expectancy increasing. By 2050, the older adult population is expected to reach 22% (1, 2). China’s aging population is particularly severe due to a large base and fast growth rate, with the older adult population projected to increase from 280 million in 2022 to 487 million in 2053. The form of population aging will become even more severe (3, 4).

The community environment serves as a critical space in shaping the daily health behaviors and social interactions of the older adult. Its high-quality spatial characteristics play a vital role in the determining the efficiency of public space utilization, determining the efficiency of public space utilization (5–8). The intensification of the aging trend has made elevated the significance of researching age-friendly communities. Responding to this, the World Health Organization (WHO) established the Global Network for Age-friendly Cities and Communities in 2010, urging nations to prioritize the construction of age-friendly communities (9). Within this context, the evaluation of age-friendly communities is recognized as a pivotal step in the construction process, serving as the foundation for implementation and guideline formulation (10). Responding to WHO’s appeal, various countries and regions have conducted research on the age-friendliness of urban and community environments based on the current situation of aging, yielding extensive outcomes. Notable examples include evaluation tools like the Community Assessment Survey for Older Adults (CASOA), the Age-Friendly Environment Assessment Tools (AFEAT), and the Elder-Friendly Urban Spaces Questionnaire (EFUSQ) (11–13), as well as evaluation guidelines like the Livable Community: An Evaluation Guide and the Age-friendly Rural and Remote Communities Guide (2007) (14, 15). In response to the challenges posed by an aging population and the growing multi-dimensional older adult care needs, China emphasizes the proactive development of age-friendly environments in community public spaces. This involves refining the assessment criteria system to comprehensively enhance the age-friendliness level of communities (16, 17). Existing research on the evaluation of age-friendly communities extensively explores the significance of evaluating indicators related to the physical and social environment in influencing the older adult (18–20). However, only a limited number of studies have elaborated on the systematic procedure of scientifically constructing evaluation systems. Yet, the scientific rigor in constructing evaluation system is fundamental to ensuring the objectivity, comprehensiveness, and authenticity of research information. This encompasses the meticulousness and feasibility of transforming research information into specific evaluation frameworks and indicators, the careful consideration of the feasibility and operability of evaluation methods, the inclusion of diverse evaluation subjects, and the determination of the evaluation process (21). Furthermore, the formulation of evaluation systems is greatly influenced by objective factors such as the built environment, economic status, and policy environment of a country or region (22–24). However, there is limited exploration of the differences in the construction process and results of evaluation systems resulting from these variations. In China, especially, the lack of scientific research on the subjects, processes, and methods of evaluation system construction, coupled with a focus on singular aspects like older adult care service facilities and operational management (25, 26), has hindered the development of a systematic and comprehensive community age-friendliness evaluation system (27–29). Therefore, there is a clear need for conducting systematic research on the assessment of age-friendly living environments for the older adult, as well as the establishment of technical standards and evaluation systems for age-friendly residential areas.

As the spatial implementation carrier of the Chinese government’s social security policy in the housing sector, the large-scale construction of affordable housing communities in Shenzhen has effectively addressed the housing issues of a significant portion of migrant families and accompanying older adult individuals, primarily from low-income groups, within the context of a mega-city (30). However, with the intensification of population aging, delayed research on policy and regulatory systems, as well as the exacerbation of social stratification and spatial differentiation, prominent contradictions have emerged within the age-friendliness configuration and environment construction in Shenzhen’s affordable housing communities. These contradictions manifest in insufficient scale of age-friendly facilities and an inadequacy in aligning age-friendly environments with the diverse needs of the older adult population, which deviates from the transition of housing development from “adequate housing” to “livable housing.” The construction of an age-friendliness evaluation system for affordable housing communities not only objectively and comprehensively reflects the degree of older adult friendliness in the community environment but also assists decision-makers in identifying intervention priorities and timelines. It enables a nuanced understanding of community strengths and weaknesses, providing a crucial tool for addressing deficiencies and guiding actions in the current context of age-friendly construction in affordable housing communities (29, 31).

The aim of this study is to systematically review the evaluation system for age-friendly communities, clarifying the scientific and systematic construction of the main subjects, evaluation methods, evaluation process, indicators and framework. Subsequently, based on the diverse needs of various stakeholders such as the older adult and the reality of public space construction in affordable housing areas in Shenzhen, a comprehensive evaluation system for age-friendliness in affordable housing communities is constructed. The feasibility and effectiveness of the evaluation system are validated through practical case studies, providing a quantitative evaluation tool for community practice and actionable guidance for policymakers. The ultimate goal is to contribute to the sustainable development of communities and effectively and judiciously meet the demands of an aging population. Additionally, in order to expand the applicability of the evaluation system construction process and address its specificity, specific policies and contexts will be taken into account during the analysis. Specifically, this study aims to explore the following questions:

How can a scientific evaluation system of age-friendly be constructed?

How can the effectiveness of the evaluation system be tested?

How can the evaluation results obtained from the evaluation tool be used to further guide the design and improvement of the age-friendly environment in public spaces of the community?

## 2 Methods

### 2.1 Literature review

The construction of age-friendly communities is a dynamic, complex, and multidimensional process. An evaluation system that is effective, reliable, and sensitive is needed to accurately measure changes in the age-friendliness of community development and provide a scientific evaluation process and outcomes for renovated and newly constructed community environments. Existing research has focused on the evaluation process, evaluation content, evaluation subjects, and evaluation methods.

#### 2.1.1 Evaluation process of age-friendly communities

**Figure 1:**
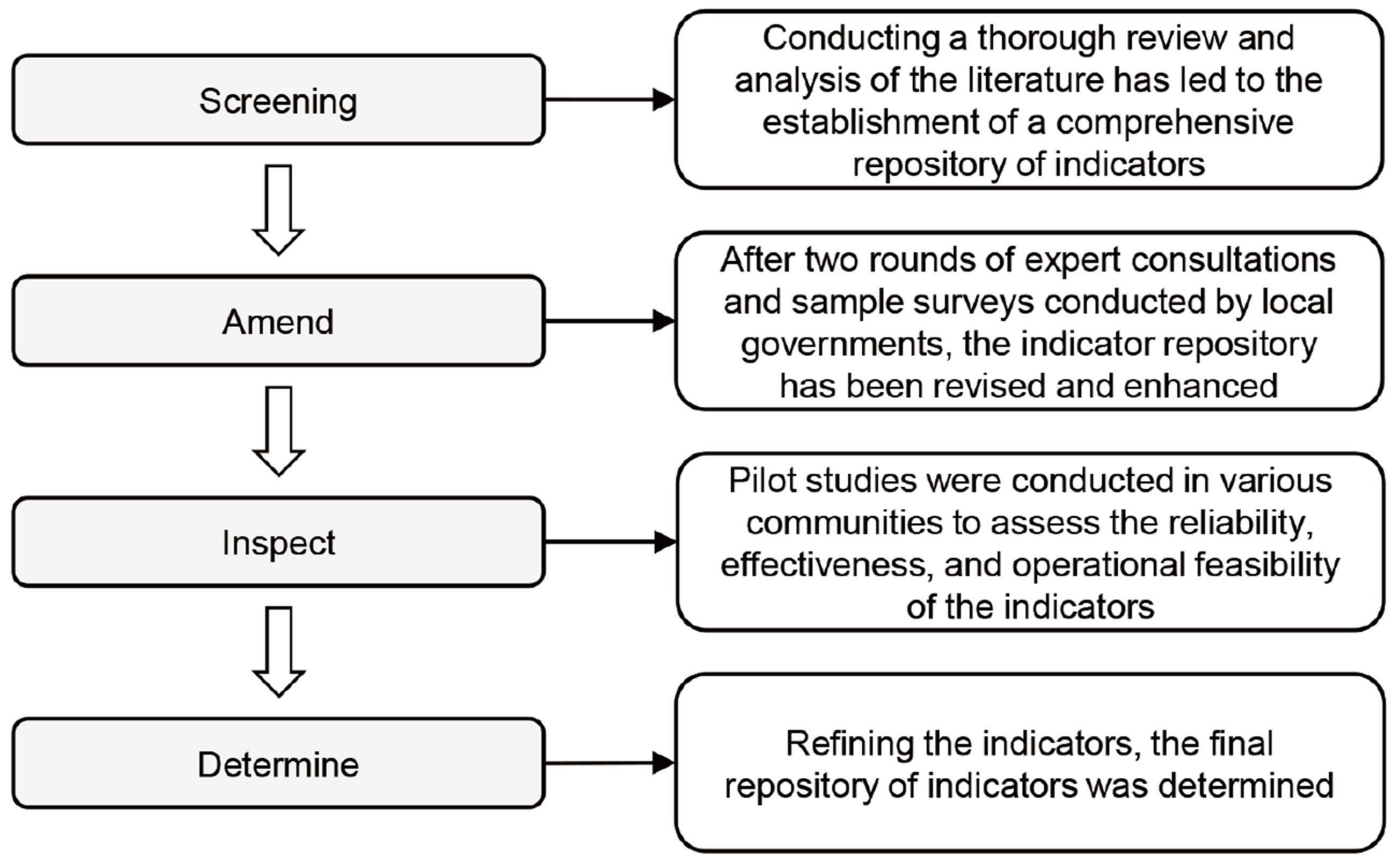
Development process of the WHO age-friendly community evaluation system.

The WHO, in an effort to expand global attention and provide an objective measure for evaluating the age-friendliness of communities in physical and social environments, developed an assessment tool for the core framework of age-friendly cities and communities from 2012 to 2014. The tool’s development underwent an extensive iterative process, including literature reviews, expert consultations, peer reviews, and pilot studies, which can be divided into four steps: (1) Screening, involving the identification of indicators through literature review; (2) Amend, entailing two rounds of international expert consultations and local government sample surveys to improve the indicator list; (3) Inspect, where pilot studies in different cities were conducted to examine the reliability and validity of the measurement of indicators; and (4) Determine, culminating in the determination of the definitive indicator list (Figure 1). This rigorous development process not only ensured the effectiveness of the tool but also provided guidance for using indicators to assess age-friendliness. The resulting “Measuring the Age-friendliness of Cities: A Guide to Using Core Indicators” has been widely adopted in various countries and regions and can serve as a reference for the construction of evaluation systems. Moreover, countries and regions have made differentiated modifications based on local needs, capabilities, and methodologies (32–35).

#### 2.1.2 Evaluation frameworks and indicators of age-friendly communities

Evaluation frameworks and indicators constitute the main content of an evaluation system. In 2007, the WHO developed the “Global Age-friendly Cities: A Guide” as a guiding evaluation tool for older people (36, 37). This guide covers eight domains of physical and social environments: outdoor spaces and buildings, transportation, housing, social participation, respect and social inclusion, civic participation and employment, communication and information, and community support and health services, which are further divided into 41 indicators. Subsequently, countries and regions have made varying modifications based on their economic development, demographic structure, policies, regulations, and other factors (14, 15, 38, 39). For example, the Lifetime Community Program in the UK focuses on seven domains, jointly published by the Department for Communities and Local Government (DCLG) and the International Longevity Centre-UK (40). The Rural and Remote Seniors Housing Guide in Canada, released in 2007 by the federal Public Health Agency of Canada and federal/provincial/territorial ministers, includes 47 indicators across eight domains (15). Compared to comprehensive and representative evaluation systems at the national and regional levels, scholars’ evaluations of specific characteristics of age-friendly communities tend to focus on certain attributes within the physical or social environment of the community (41, 42).

#### 2.1.3 Evaluation subjects of age-friendly community

The multi-stakeholder evaluation of age-friendly communities is a growing trend, which involves establishing partnerships between organizations and individuals with different natures such as government agencies and non-governmental organizations. It develops a combined “top-down” and “bottom-up” participation model, expands the scope of organizations concerned with aging, and shares development achievements widely (43–45). Among them, older adults express their needs and participate in affairs from the “bottom-up” as direct beneficiaries (46), social organizations participate in community governance and contribute to activating diverse forces at the grassroots level (47), and the government promotes the establishment of cooperative partnerships through policy tools, funding support, and resource coordination (48). All parties systematically focus on aging issues from different perspectives and levels (49, 50). For example, the collaborative model of “University-Community-Government” in the United States is a representative of the bottom-up approach. Through universities initiating project development of indicator libraries, participants such as older adults and community organizations are involved in revising the indicator library, and government personnel have the final decision-making power and provide support such as funding assistance. Interdisciplinary discussions and cooperation are key to the sustainable development of age-friendly communities (44). Interdisciplinary discussions and cooperation are key to the sustainable development of age-friendly communities.

#### 2.1.4 Evaluation methods of age-friendly community

When evaluating tools and methods, two types of evaluation are currently used: qualitative and quantitative. Qualitative evaluation involves the use of methods such as questionnaires, focus groups, multi-party interviews, and on-site research. These methods provide intuitive insights into detailed information about participants, measure the needs and perceived environment of older adult individuals in the community, and offer scientific data and evidence for targeted solutions (51, 52). For instance, in 2007, the WHO held focus groups and interviewed older adult during the research and design process of developing indicators for age-friendly cities and communities (10, 53). However, qualitative methods involve a process of studying by obtaining subjective feelings of the target population, which leads to drawbacks such as complex sample selection processes, susceptibility to subjective misleading of participants, and lengthy time consumption (54). Quantitative research methods obtain specific properties of the environment through observations and measurements and conduct descriptive and objective data analysis. They are generally used in the data analysis process to evaluate differences in demographic characteristics and the perceived impact of older adult health and quality of life on the degree of age-friendliness (55, 56). The subjective perception evaluation of indicators by older adult, government officials, nursing staff, and other stakeholders forms the basis of both qualitative and quantitative methods. Currently, research lacks actual objective data measurement and quantitative evaluation of construction content. Dellamora et al. (10) and Liu et al. (52) also found a lack of accurate and operational methods to measure the characteristics of age-friendly communities. Therefore, future research can combine qualitative and quantitative methods to correct errors caused by single evaluation methods, improve accuracy, and provide technical support for the effective application of the evaluation system.

In conclusion, constructing an effective evaluation system for age-friendly communities necessitates a meticulous process encompassing screening, amendment, inspection, and determination. Collaborative engagement with diverse stakeholders, such as government bodies, the older adult, and social organizations, is crucial. Defining stratified evaluation criteria and employing a mix of quantitative and qualitative assessment methods are key elements.

### 2.2 Study design

The analysis of existing literature reveals that effective evaluation processes, collaborative relationships among multiple stakeholders, hierarchical evaluation content, and diverse evaluation methods are the primary factors influencing the development of an age-friendly evaluation system. Moreover, by considering the unique context of Shenzhen’s affordable housing policy, the state of age-friendly construction in residential areas, and the specific needs of older adult individuals, the evaluation system can effectively account for regional variations.

**Figure 2:**
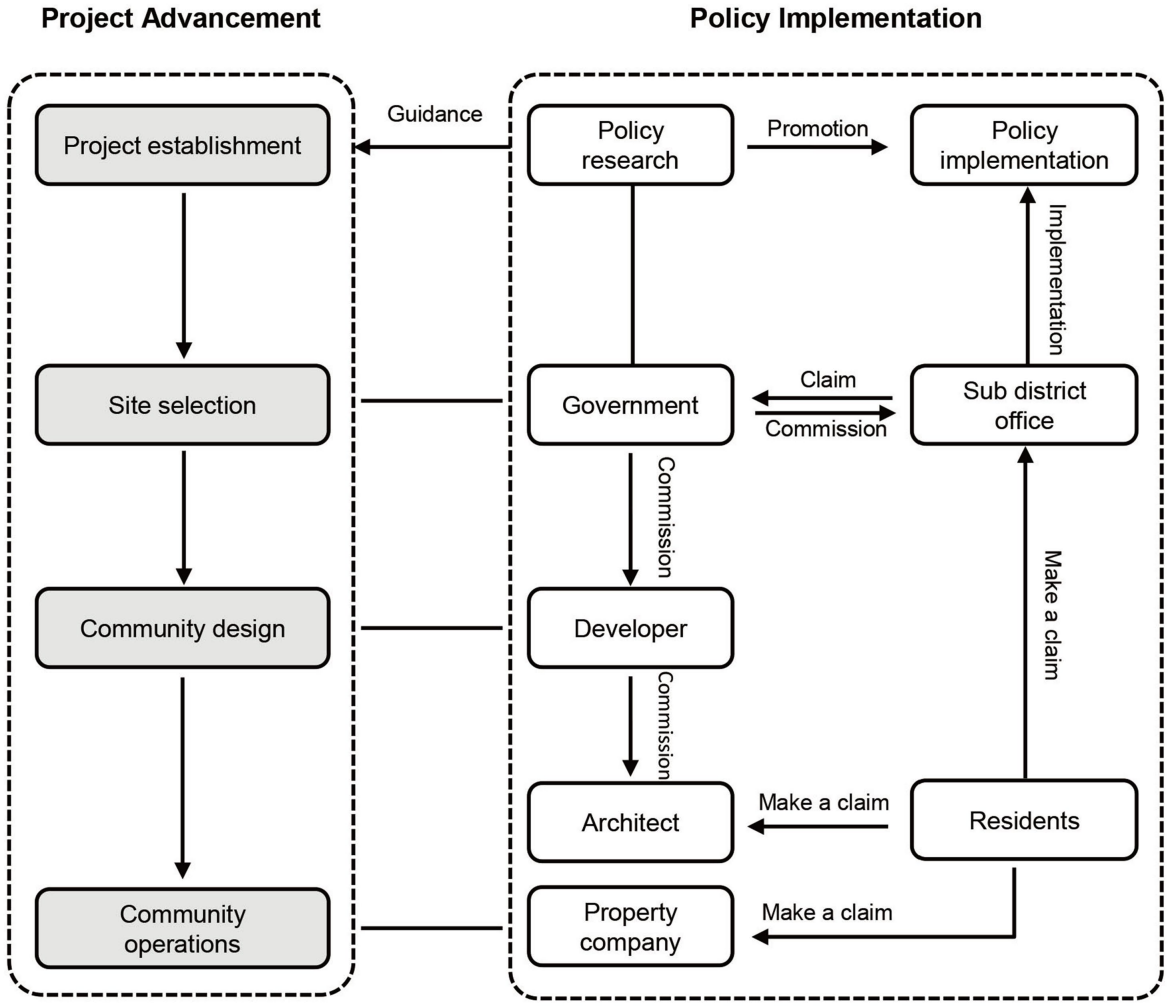
The process of advancing multiple subjects in affordable housing community. Adapted with permission from (58).

To address the aforementioned research questions, the research encompasses three key stages: the selection of age-friendly evaluation content, the construction of the evaluation system, and the validation of its effectiveness. The process of selecting evaluation content follows the evaluation tool development framework established by the WHO, which involves four sequential steps: preliminary selection, screening, amend, and determine. Additionally, the development of age-friendly features within Shenzhen’s affordable housing communities involves the active participation of various stakeholders, including government agencies, residents, designers, and others (57, 58) (Figure 2). Consequently, due to the unique policy context, the evaluation subjects primarily comprise the users of public spaces within affordable housing communities—the older adult, policymakers and researchers involved in age-friendly initiatives—government departments, architects and planners responsible for designing public spaces, as well as policy implementers such as sub district office and community workers. This collaborative model, combining both “top-down” and “bottom-up” approaches, allows for comprehensive consideration of age-friendly issues in Shenzhen’s affordable housing communities from diverse perspectives, thus ensuring the targeted development of the evaluation system. Given the subjective and objective nature of the research process, we have adopted qualitative methods, including questionnaire surveys and interviews, to analyze the perceptions and evaluations of various stakeholders regarding the age-friendly outdoor environments within affordable housing communities. Moreover, quantitative methods such as factor analysis and AHP have been employed to explore the intricate and varied indicators and coefficients.

**Figure 3:**
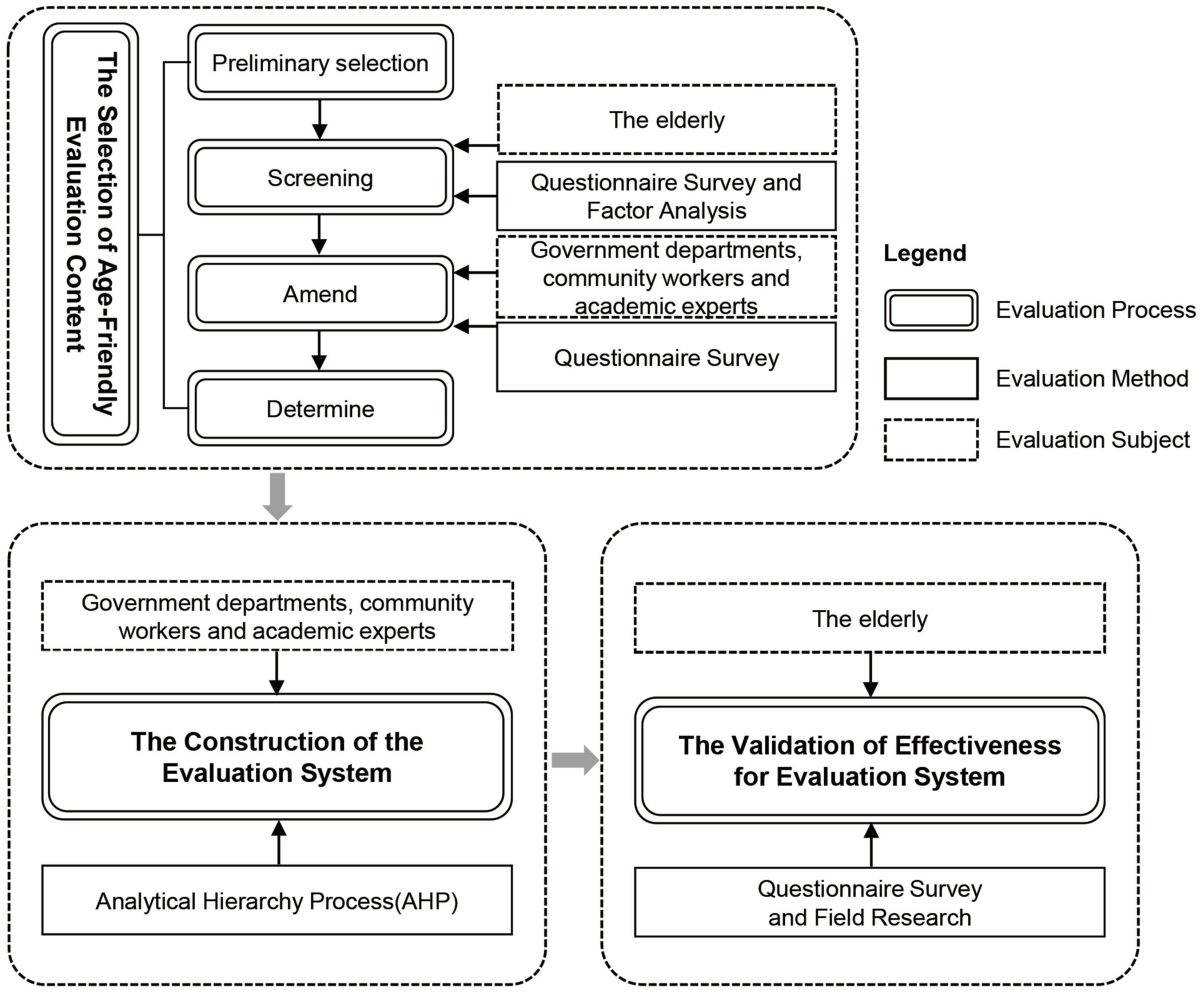
Structure of the proposed frameworks in this study.

The overall conceptual framework is depicted below (Figure 3).

## 3 Results

### 3.1 Site selection

**Figure 4:**
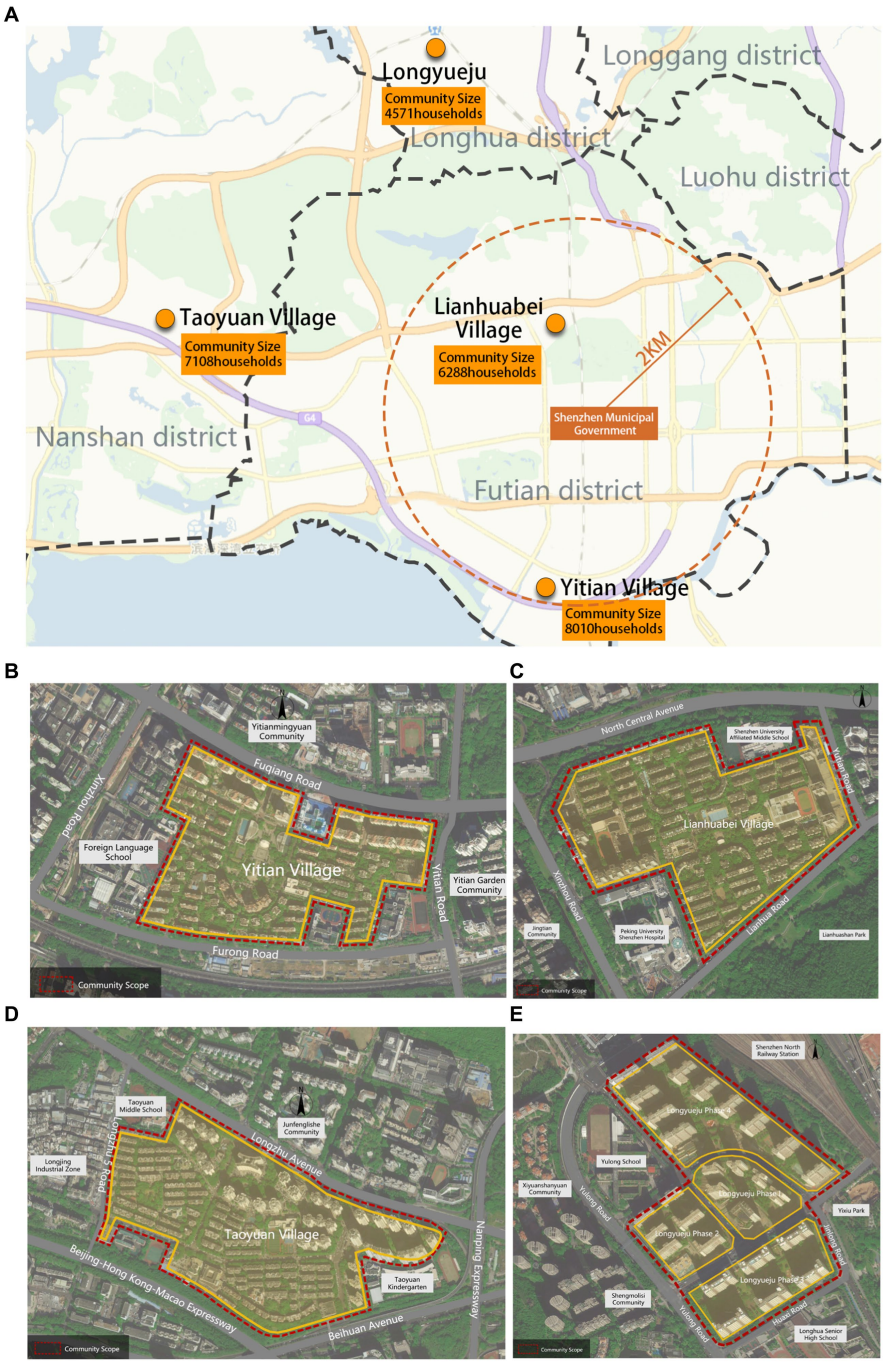
Community location maps. **(A)** The spatial distribution of the four communities. **(B)** Location of the Yitian Village. **(C)** Location of the Lianhuabei Village. **(D)** Location of the Taoyuan Village. **(E)** Location of the Longyueju.

More than 30 years, the development of affordable housing in Shenzhen has undergone distinct phases influenced by national policies and urban developmental stages. These stages, shaped by policy shifts and construction approaches, can be categorized into five phases: the exploration of housing systems, the implementation of housing systems, the exploration of affordable housing policies, comprehensive affordable housing construction, and the concurrent development of talent housing and general housing security (59, 60). Each stage exhibits significant variations in the construction area, spatial characteristics, and demographic composition of affordable housing communities. This research adheres to the following selection principles: (1) Inclusion of affordable housing communities that align with the developmental patterns of housing in Shenzhen and demonstrate relative maturity; (2) Ensuring diversity in terms of demographic composition and construction timelines, encompassing both migrant and local older adult residents; (3) Prioritizing communities with relatively high population densities of older adult individuals to ensure the reliability and validity of evaluation data. Ultimately, the four selected communities for study are Taoyuan Village, Lianhuabei Village, Yitian Village, and Longyueju (Table 1 and Figure 4).

**Table 1:** Basic information on the four selected affordable housing communities.

Community name	Lianhuabei Village	Taoyuan Village	Yitian Village	Longyueju
Community location	Futian district	Nanshan district	Futian district	Longhua district
Completion time	1994	1997 2000 2008	1994	2011
Construction background	Subsequent implementation of the housing system	Exploration of affordable housing policies	Subsequent implementation of the housing system	Concurrent development of talent housing and general housing guarantee
Percentage of older adult	12.61%	15%	10.3%	9.7%

### 3.2 The selection of age-friendly evaluation content

The hierarchical evaluation content comprises primary and secondary assessment indicators. With the primary indicators aimed at accurately describing the current situation and problem categories. For this study, the primary indicators were derived from representative policy documents and regulatory standards at both the national and Shenzhen city levels in China. These documents, including relevant policy directives guiding age-friendly development, exhibit a forward-looking and open-ended nature (61), while the regulatory standards offer operational guidelines (62–64). The synergistic integration of these two sources serves as the foundation for constructing the evaluation system. Consequently, four elements—activity spaces, road traffic, service facilities, and green environment—are selected as the primary assessment framework for this study. The selection of secondary assessment indicators requires a detailed breakdown of each environmental factor, ensuring the completeness, diversity, and controllability of each assessment element. This process followed four steps: initial selection, screening, amend, and final determination.

#### 3.2.1 Preliminary selection of indicators

The selection of secondary indicators adhered to the principles of comprehensive coverage and localization characteristics. The screening of indicators drew from two sources: (1) Policy documents and regulatory standards in Guangdong Province (65, 66); (2) Relevant literature on age-friendly community evaluations. The literature screening process involved searching the CNKI and Web of Science databases with the keywords “age-friendly community” and “evaluation.” This yielded 82 highly cited and relevant papers with a high impact factor are analyzed. The evaluation indicators and their frequency of appearance were analyzed, resulting in the formulation of a representative technical framework and evaluation elements. Ultimately, a total of 35 secondary indicators were obtained (Table 2).

**Table 2:** The preliminary selection of indicators.

Primary evaluation indicators	Secondary evaluation indicators
Activity spaces	Adequacy of activity spaces, Diversity of activity space types, Rational division of activity spaces, Accessibility of activity spaces, Proportion of parent–child spaces, Proportion of spaces dedicated to the older adult, Transparency of spatial sightlines
Green landscape	Safety of plant species, Seasonal color variation of plants, Aesthetic appeal of plant landscapes, Affinity of plant landscapes, Proportion of therapeutic plant species, Aesthetic quality of water features
Service facilities	Configuration of emergency facilities, Placement of signage facilities, Clarity of signage facilities, Installation of lighting facilities, Night illumination level of lighting facilities, Diversity of recreation facilities, Safety of recreation facilities, Usability of recreation facilities, Comfort of recreation facilities, Placement of sanitation facilities, Provision of resting facilities, Comfort of resting facilities, Completeness of accessibility facilities, Adequacy of safety and protection facilities, Configuration of sunshade facilities
Road and transportation	Safety of transportation, Completeness of vehicle roadway facilities, Rate of vehicular encroachment on road, Provision of accessible parking spaces, Safety of pedestrian pathways, Accessibility of pedestrian pathways, Comfort of pedestrian pathways

#### 3.2.2 Screening of indicators

The process of screening evaluation indicators is primarily driven by the needs of the older adult, engaging their expressed preferences and participation in activities to reflect an objective perception.

This involves a “bottom-up” screening of initial indicators. The approach employed face-to-face interviews through a questionnaire survey, targeting the older adult, with the preliminary selection of 35 secondary evaluation indicators and open-ended questions forming the questionnaire content. Taking into account factors such as older adult’s outdoor activity times, behavioral patterns, and the climate characteristics of Shenzhen, the survey was conducted during three time periods: 6:30–7:30 am, 9:00–11:00 am, and 4:00–6:30 pm. A total of 186 formal questionnaires were distributed among four communities (Table 3), with 183 valid questionnaires being entered and coded for analysis.

**Table 3:** Basic information on the questionnaire for the screening indicators.

Population studied	Location of the research	Duration of the research (2022)	Number of questionnaires
Older and soon-to-be older age groups (age ≥ 55)	Taoyuan Village	06.03–06.05, 06.07–06.08, 06.13–06.14	45
	Lianhuabei Village	06.11–06.13, 06.15–06.16	50
	Yitian Village	06.11, 06.15–06.16	41
	Longyueju	06.18–06.19, 6.21	50

Factor Analysis was employed, facilitating the aggregation of overlapping indicators into a few independent common factors through correlation analysis, thereby verifying the accuracy of the initial indicator classification (67). To begin, in testing the number of common factors, four primary indicators were used as a fixed number of factors, and the 35 secondary indicators were categorized. The results indicated the extraction of four common factors with eigenvalues greater than 1, explaining a cumulative variance of 77.137%. This suggests the good validity of the four primary indicators in the preliminary selection process (Table 4). Subsequently, to assess the correspondence between the secondary and primary indicators, factor analysis was performed on the secondary indicators, resulting in a rotated component matrix. Indicators with loadings less than 0.5 were eliminated, resulting in the screening selection of indicators (Table 5), including four primary indicators and 30 secondary indicators. However, it is important to note that due to the significant impact of affordable housing construction and the subjective nature of older adult needs, the indicator selection process has limitations. Further adjustments to the indicators are therefore necessary.

**Table 4:** Variable interpretation.

Component	Initial eigenvalues			Total extracted loadings squared			Rotated loadings squared		
	Total	Percentage of variance	Cumulative %	Total	Percentage of variance	Cumulative %	Total	Percentage of variance	Cumulative %
1	17.881	51.090	51.090	17.881	51.090	51.090	8.857	25.307	25.307
2	5.406	15.447	66.536	5.406	15.447	66.536	7.987	22.821	48.128
3	2.392	6.835	73.371	2.392	6.835	73.371	6.930	19.800	67.928
4	1.318	3.765	77.137	1.318	3.765	77.137	3.223	9.209	77.137

**Table 5:** Adjustment of indicators.

Indicators with loadings<0.5	Adjustment method
Rational division of activity spaces	Elimination of Indicators
Proportion of spaces dedicated to the older adult	
Safety of recreation facilities	
Usability of recreation facilities	
Comfort of recreation facilities	
Configuration of accessible parking spaces	

#### 3.2.3 Amendment of indicators

The amendment process of indicator relies on multiple stakeholders including government authorities, community workers, and academic experts, who contribute “top-down” evaluation information to adjust the selected indicators. Government departments and community workers are responsible for the construction and management of affordable housing communities, driving age-friendly development through policy formulation, financial support, and regulatory oversight. Academic experts, engaged in residential design research, address the age-friendly needs of the older adult in housing environments, evaluating the characteristics of residential areas with a high level of expertise. They play a crucial role in ensuring a fair and objective selection of indicators.

The research methodology involved a questionnaire survey to assess the screening indicators. A total of 25 participants, including government officials, experts, and community workers, took part in the survey. Based on the previously selected indicators, a questionnaire was administered to the experts, yielding four types of correction results: deletion of indicators, addition of indicators, modification of indicators, and consolidation of indicators (Table 6).

**Table 6:** Amended results of indicators.

Amend result	Specific amendment items
Deletion of indicators	Seasonal color variation of plants
	Diversity of recreation facilities
Addition of indicators	Safety of activity spaces
	Green shade ratio
	Placement of outdoor storage shelves
Modification of indicators	Splitting “Affinity of plant landscapes” into “Accessibility of plant landscapes” and “Cultural aspects of plant landscapes”
	Splitting “Aesthetic quality of water features” into “Aesthetic cleanliness of water features” and “Functionality of water features”
	Amend “Completeness of vehicle roadway facilities” into “Adequacy of crossing facility”
Consolidation of indicators	Combining “Adequacy of safety and protection facilities” and “Completeness of accessibility facilities” into “Completeness of accessibility facilities”
	Combining “Rate of vehicular encroachment on road” and “Accessibility of pedestrian pathways” into “Accessibility of pedestrian pathways”

#### 3.2.4 Determination of indicators

Through the process of selecting and amending the predetermined set of evaluation indicators, 4 primary indicators and 30 secondary indicators were ultimately determined for the age-friendliness evaluation system of public spaces in affordable housing communities in Shenzhen (Table 7).

**Table 7:** Predefined set of evaluation indicators.

Evaluation objective	Primary indicators	Secondary indicators
The Age-Friendly Evaluation System of Public Spaces in Affordable Housing Communities in Shenzhen	Activity Space	Adequacy of activity spaces
		Diversity of activity space types
		Accessibility of activity spaces
		Proportion of parent–child spaces
		Transparency of spatial sightlines
		Safety of activity spaces
	Green Landscape	Safety of plant species
		Cultural aspects of plant landscapes
		Aesthetic appeal of plant landscapes
		Accessibility of plant landscapes
		Proportion of therapeutic plant species
		Green shade ratio
		Aesthetic cleanliness of water features
		Functionality of water features
	Service Facilities	Configuration of emergency facilities
		Placement of signage facilities
		Clarity of signage facilities
		Installation of lighting facilities
		Night illumination level of lighting facilities
		Placement of sanitation facilities
		Provision of resting facilities
		Comfort of resting facilities
		Completeness of accessibility facilities
		Configuration of sunshade facilities
		Placement of outdoor storage shelves
	Road and Transportation	Safety of transportation
		Adequacy of crossing facility
		Safety of pedestrian pathways
		Accessibility of pedestrian pathways
		Comfort of pedestrian pathways

### 3.3 The construction of the evaluation system

The construction of the evaluation framework is based on the aforementioned assessment criteria. This process involves the quantification of the age-friendliness of the community through the AHP, encompassing the establishment of a hierarchical structure, weight calculation, and consistency verification.

#### 3.3.1 Modeling the hierarchical structure of the evaluation indicators

**Figure 5:**
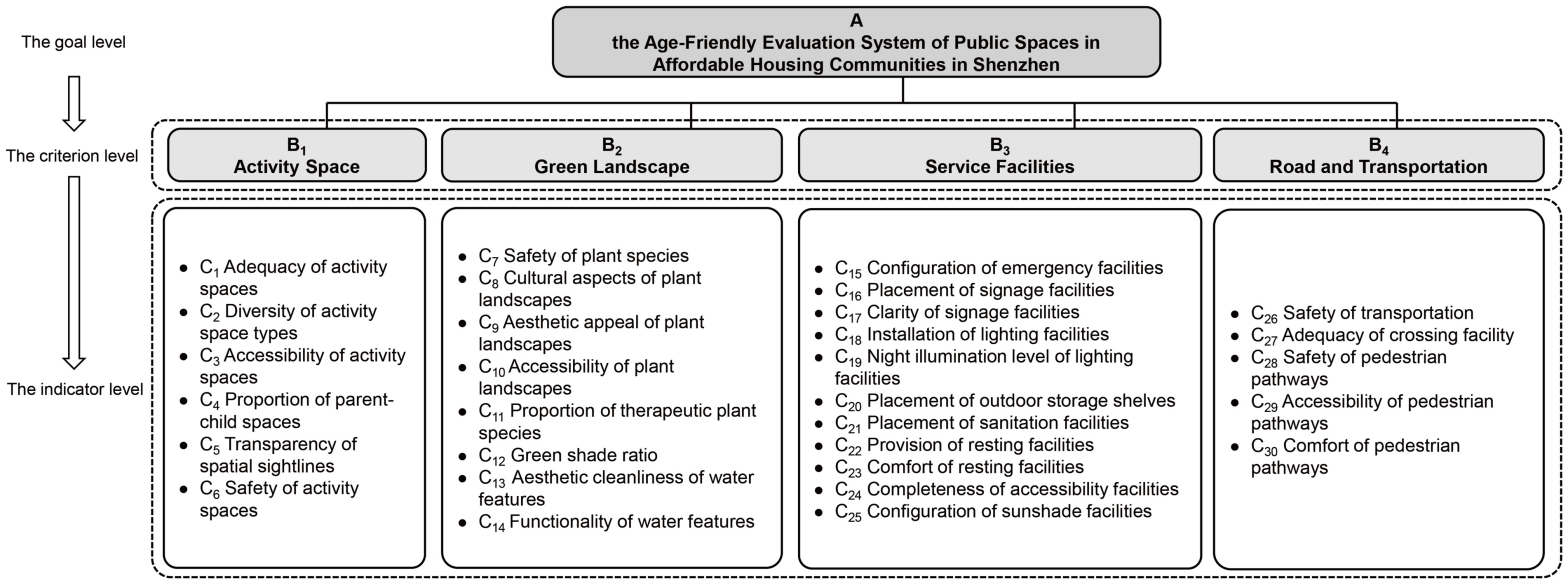
Hierarchical structure model for the age-friendly evaluation of public spaces in Shenzhen’s affordable housing communities.

The establishment of the hierarchical structure model involves the process of hierarchically organizing evaluation objectives into three parts: the goal level (A), criterion level (B), and indicator level (C) (Figure 5). Utilizing Professor T.L. Saaty’s “1–9” importance rating scale, each indicator within each level is comparatively assessed through pairwise comparisons, generating a comparative judgment matrix to determine the weights of the respective indicators (68). In the research process, a survey questionnaire was distributed to 25 diverse evaluators to obtain their assessments and ratings on the weights of the evaluation indicators. In order to avoid data errors caused by subjective ideas, the Grubbs Criterion was used to screen the valid data and homogenization was done.

#### 3.3.2 Calculation of evaluation indicator weights and consistency tests

Weighting of age-friendly indicators reflects the varying degrees of importance assigned to indicators at different levels within the evaluation system, which affects the evaluation results of the community’s aging-friendliness. An arithmetic-average method was utilized to calculate the root vectors of each indicator. This was followed by column normalization, and the eigenvectors of the judgment matrix were calculated, representing the weight values of the indicators at each level within the evaluation system. As an illustration, the weights of the primary indicators are presented in Table 8.

**Table 8:** Results of weighting of primary indicators.

Comparison value (*B*_ij_)	Activity space	Green landscape	Service facilities	Road and transportation	Weights
Activity space	1.000	2.959	0.986	2.179	36.165%
Green landscape	0.338	1.000	0.986	0.490	10.949%
Service facilities	1.014	2.988	0.335	1.129	31.007%
Road and transportation	0.459	2.042	1.000	1.000	21.880%

Due to the abundance of age-friendly evaluation indicators, discrepancies may arise when various stakeholders compare and assign values to these indicators. To rectify this bias, a consistency test is required. The testing procedure encompasses determining the maximum eigenvalue of the judgment matrix, calculating the consistency index, searching for the consistency index, and computing the consistency ratio. Taking the primary indicators as an example (Table 9).

**Table 9:** Consistency test results of primary indicators.

Consistency test	Eigen vector	Weights	λmax	CI	RI	CR
Activity space	1.447	36.165%	4.043	0.014	0.890	0.016
Green landscape	0.438	10.949%				
Service facilities	1.240	31.007%				
Road and transportation	0.875	21.880%				

Ultimately, the weight coefficients for each indicator factor in Shenzhen’s affordable housing community public space evaluation system and the corresponding target weight ratios are derived, as demonstrated in Table 10.

**Table 10:** Weights of evaluation indicators for age-friendly of public space in Shenzhen’s affordable housing communities.

Goal level (A)	Criteria level (B)	Weight of primary indicator	Indicator level (C)	Weight of secondary indicator	Weight of secondary indicators to goal
(A) The Age-Friendly Evaluation System of Public Spaces in Affordable Housing Communities in Shenzhen	(B_1_) Activity Space	0.362	C_1_ Adequacy of activity spaces	0.192	6.95%
			C_2_ Diversity of activity space types	0.058	2.10%
			C_3_ Accessibility of activity spaces	0.135	4.89%
			C_4_ Proportion of parent–child spaces	0.157	5.68%
			C_5_ Transparency of spatial sightlines	0.081	2.93%
			C_6_ Safety of activity spaces	0.378	13.68%
	(B_2_) Green Landscape	0.110	C_7_ Safety of plant species	0.321	3.53%
			C_8_ Cultural aspects of plant landscapes	0.031	0.34%
			C_9_ Aesthetic appeal of plant landscapes	0.101	1.11%
			C_10_ Accessibility of plant landscapes	0.059	0.65%
			C_11_ Proportion of therapeutic plant species	0.083	0.91%
			C_12_ Green shade ratio	0.287	3.16%
			C_13_ Aesthetic cleanliness of water features	0.059	0.65%
			C_14_ Functionality of water features	0.060	0.66%
	(B_3_) Service Facilities	0.310	C_15_ Configuration of emergency facilities	0.198	6.14%
			C_16_ Placement of signage facilities	0.016	0.50%
			C_17_ Clarity of signage facilities	0.016	0.50%
			C_18_ Installation of lighting facilities	0.071	2.20%
			C_19_ Night illumination level of lighting facilities	0.071	2.20%
			C_20_ Placement of outdoor storage shelves	0.028	0.87%
			C_21_ Placement of sanitation facilities	0.045	1.40%
			C_22_ Provision of resting facilities	0.143	4.43%
			C_23_ Comfort of resting facilities	0.04	1.24%
			C_24_ Completeness of accessibility facilities	0.272	8.43%
			C_25_ Configuration of sunshade facilities	0.102	3.16%
	(B_4_) Road and Transportation	0.219	C_26_ Safety of transportation	0.207	4.53%
			C_27_ Adequacy of crossing facility	0.104	2.28%
			C_28_ Safety of pedestrian pathways	0.296	6.48%
			C_29_ Accessibility of pedestrian pathways	0.244	5.34%
			C_30_ Comfort of pedestrian pathways	0.150	3.29%

### 3.4 Validation of the effectiveness of the evaluation system

In general, an evaluation system is a method for qualitatively or quantitatively measuring things. The results can specifically indicate the direction and extent of changes in certain things during a specific period, in order to improve their sustainability in the future (69). The validation of the effectiveness of the evaluation system involves applying the constructed evaluation system to selected affordable housing communities, assessing its applicability as a tool. This study specifically chose Lianhuabei Village (welfare housing) and Longyueju (talent housing) in Shenzhen as case communities. They differ in terms of construction time and target beneficiaries, representing typical and representative types of affordable housing in Shenzhen. Utilizing an age-friendly evaluation system as a foundation, survey questionnaires were designed. To ensure sample uniformity and rationality, public spaces with evenly distributed older adult and diverse activities were surveyed in both communities, with 50 valid questionnaires collected from each community. After the reliability and validity of the questionnaire results were tested, the scores for each indicator were first obtained using the average value method. Subsequently, each indicator score was multiplied by its corresponding weight value from Table 10, and the results were summed to obtain the final score. Finally, the quantitative standards and grades of the Likert scale were utilized to form the overall evaluation results (Table 11).

**Table 11:** Results of the evaluation of aging in the studied communities.

Name	Score results					Age-friendly evaluation	Evaluation results
	Activity space	Green landscape	Service facilities	Road and transportation	Total score		
Longyueju	1.079	0.360	0.939	0.902	3.246	2.5<*X*_j_ ≤ 3.5	General
Lianhuabei Village	1.473	0.356	1.144	0.593	3.016		

**Figure 6:**
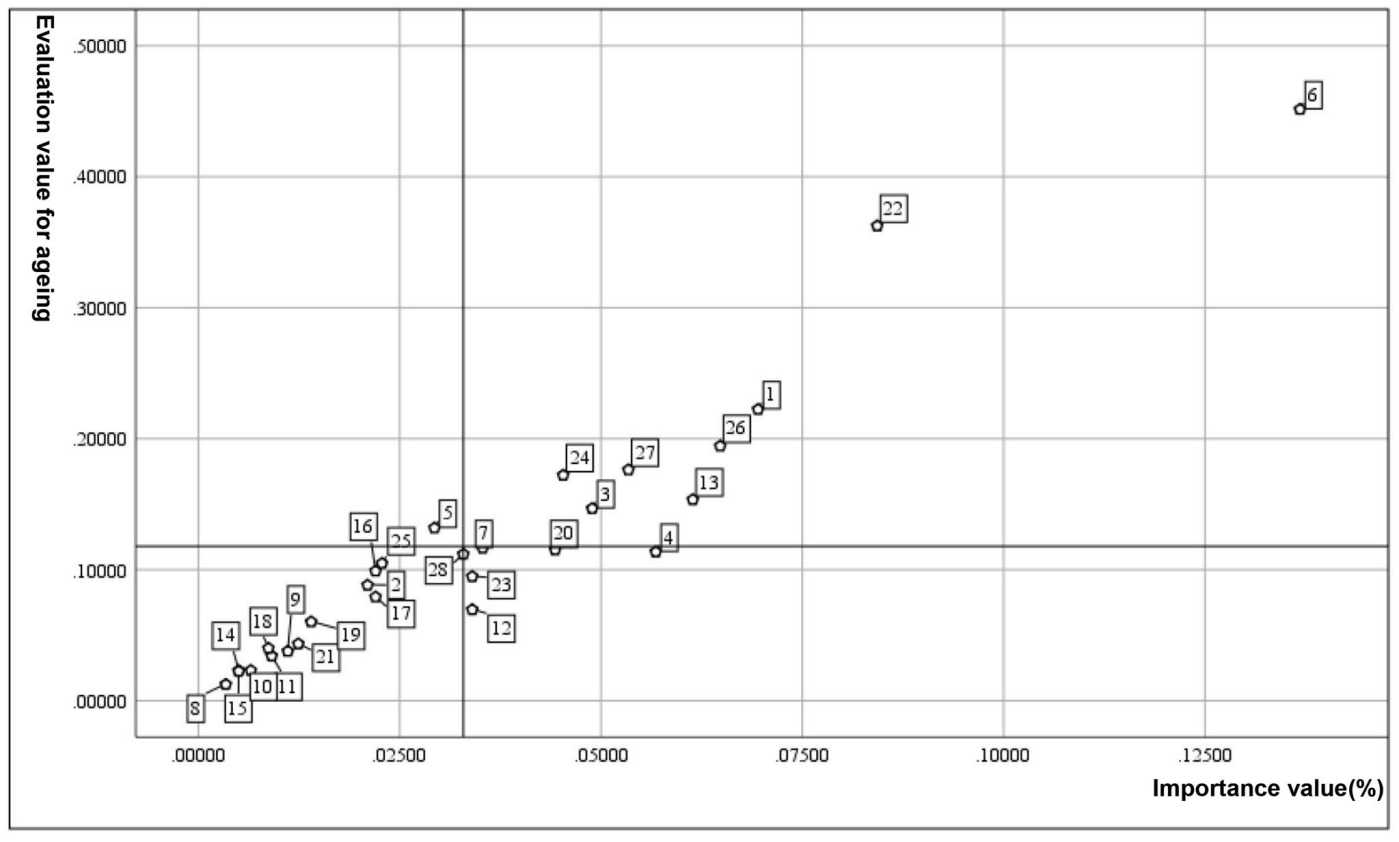
Quadrant analysis of Longyueju evaluation values. C_1_, adequacy of activity spaces; C_2_, diversity of activity space types; C_3_, accessibility of activity spaces; C_4_, proportion of parent–child spaces; C_5_, transparency of spatial sightlines; C_6_, safety of activity spaces; C_7_, safety of plant species; C_8_, cultural aspects of plant landscapes; C_9_, aesthetic appeal of plant landscapes; C_10_, accessibility of plant landscapes; C_11_, proportion of therapeutic plant species; C_12_, green shade ratio; C_13_, configuration of emergency facilities; C_14_, placement of signage facilities; C_15_, clarity of signage facilities; C_16_, installation of lighting facilities; C_17_, night illumination level of lighting facilities; C_18_, placement of outdoor storage shelves; C_19_, placement of sanitation facilities; C_20_, provision of resting facilities; C_21_, comfort of resting facilities; C_22_, completeness of accessibility facilities; C_23_, configuration of sunshade facilities; C_24_, safety of transportation; C_25_, adequacy of crossing facility; C_26_, safety of pedestrian pathways; C_27_, accessibility of pedestrian pathways; C_28_, comfort of pedestrian pathways.

**Figure 7:**
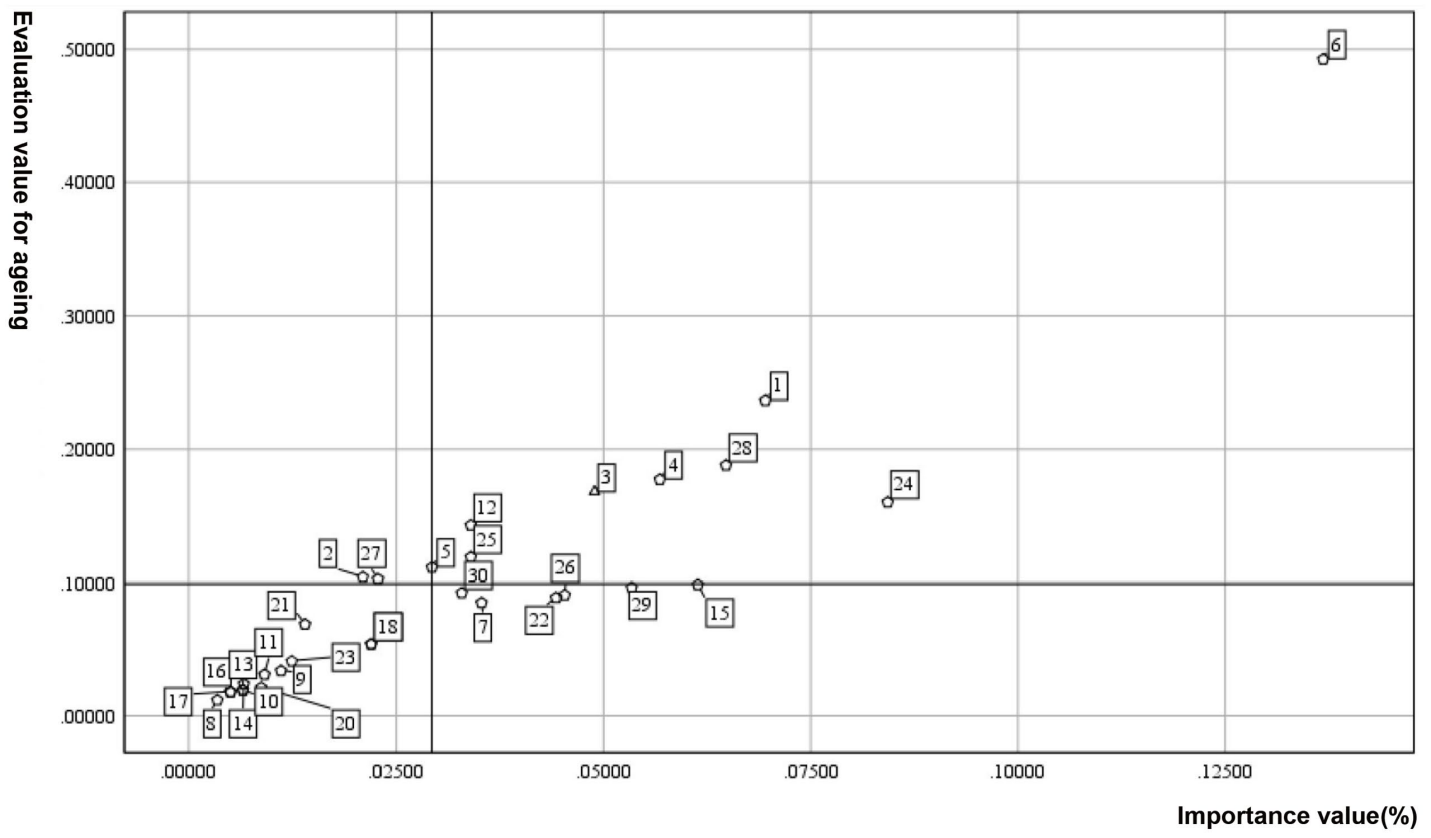
Quadrant analysis of Lianhuabei Village evaluation values. C_1_, adequacy of activity spaces; C_2_, diversity of activity space types; C_3_, accessibility of activity spaces; C_4_, proportion of parent–child spaces; C_5_, transparency of spatial sightlines; C_6_, safety of activity spaces; C_7_, safety of plant species; C_8_, cultural aspects of plant landscapes; C_9_, aesthetic appeal of plant landscapes; C_10_, accessibility of plant landscapes; C_11_, proportion of therapeutic plant species; C_12_, green shade ratio; C_13_, aesthetic cleanliness of water features; C_14_, functionality of water features; C_15_, configuration of emergency facilities; C_16_, placement of signage facilities; C_17_, clarity of signage facilities; C_18_, installation of lighting facilities; C_19_, night illumination level of lighting facilities; C_20_, placement of outdoor storage shelves; C_21_, placement of sanitation facilities; C_22_, provision of resting facilities; C_23_, comfort of resting facilities; C_24_, completeness of accessibility facilities; C_25_, configuration of sunshade facilities; C_26_, safety of transportation; C_27_, adequacy of crossing facility; C_28_, safety of pedestrian pathways; C_29_, accessibility of pedestrian pathways; C_30_, comfort of pedestrian pathways.

The evaluation results indicate that the Lianhuabei Village and Longyueju share common characteristics, with both scoring at a generally age-friendly level. However, there are variations at the criteria level. Further, satisfaction levels and indicator weights were used to generate quadrant diagrams, resulting in four quadrants (I, II, III, and IV), representing high importance—high age-friendliness, low importance—high age-friendliness, low importance—low age-friendliness, and high importance—low age-friendliness (Figures 6, 7). Notably, indicators in the quadrant IV, reflecting high importance—low older adult-friendliness, underscore deficiencies in the older adult-friendliness of public spaces that are in high demand among the older adult, warranting focused attention. Specifically, the evaluation reveals that the Longyueju faces challenges concerning the proportion of parent–child spaces, the safety of plant species, the green shade ratio, and the provision of resting facilities. Therefore, moderate improvement is required to enhance its older adult-friendliness (Figure 6). On the other hand, the evaluation indicators for Lianhuabei Village primarily focus on the safety of plant species, the provision of resting facilities, the safety of transportation, and the accessibility of pedestrian pathways (Figure 7). To improve the level of older adult-friendliness, moderate improvement can be implemented, particularly in terms of enhancing the comfort of pedestrian pathways. The commonality and distinctiveness in the age-friendliness assessment results serve as a basis for the formulation of improvement strategies.

## 4 Discussion

The age-friendly evaluation system of community has the unique advantage of comprehensively and meticulously evaluating the built environment factors. It stratifies and grades the assessment results, providing a pathway and tool for identifying issues related to the construction of age-friendly communities (70). In this context, this study revealed the process and elements involved in constructing an assessment system. Taking Shenzhen’s affordable housing community public spaces as a case study, a collaborative effort involving the older adult, government officials, and expert scholars was undertaken. Based on the preliminary results of indicator selection, factor analysis was applied to screen the indicators, and expert questionnaires were employed to further amend them. Consequently, a comprehensive age-friendly evaluation system of public spaces in Shenzhen’s affordable housing communities was established, comprising 4 primary indicators and 30 secondary indicators. The construction of the age-friendly evaluation system by utilizing the AHP, was validated for its effectiveness and applicability. The research results indicate that the age-friendly evaluation system for affordable housing communities serves, on one hand, as a measure of the construction level of age-friendly physical space environments, providing an overview of the direction for overall age-friendliness improvement. On the other hand, through comparative studies, it reflects individual variations within the community, thereby assisting in the specific improvement of age-friendly features.

In the broader context of age-friendliness improvement, several studies indicate that despite their lower economic status, affordable housing communities exhibit commendable performance in social integration and diverse collaborative governance (71, 72). Additionally, the Shenzhen municipal government has issued multiple policies supporting the co-construction and sharing of affordable housing communities (73). Both Longyueju and Lianhuabei Village fall within the category of affordable housing communities, demonstrating a moderate level of age-friendliness in their public spaces. When considering holistic improvement for older adult-friendliness, the following principles may be considered: (1) Balancing economic efficiency and practicality by providing low-cost, high-efficiency multi-level communication spaces. This is due to the cooperative and shared construction approach in Shenzhen’s affordable housing, emphasizing the economic, functional, and practical aspects of space design during usage. Moreover, given the prevalence of non-local older adult within the housing area and the diverse demands from various demographics, there is a need to integrate existing resources strategically. By employing cost-effective strategies such as functional zoning through replacement and the incorporation of new spatial functionalities, the goal is to create public spaces finely tuned to the daily lives of the older adult, thereby fostering efficient social interactions. (2) Emphasizing diversity and participatory design is crucial for enhancing the sense of belonging among the older adult. The spontaneous “public participation” plays a pivotal role in the construction of age-friendly environments. Given the significant proportion of relocating older adult individuals in Shenzhen’s affordable housing communities, age-friendly construction should focus on the design of daily communication spaces with a direction toward humanization, diversification, and a sense of belonging.

In the context of specific age-friendliness improvement strategies, community-specific considerations are crucial. The research indicates significant divergences in age-friendliness assessments for activity spaces and road traffic between the two communities. Two primary factors contribute to these disparities: (1) Varied family structures result in distinct needs. Longyueju, representing talent housing, exhibits 78% multi-generational family structures, while Lianhuabei Village, as a welfare housing example, shows more diverse family structures with 32% multi-generational and 40% living with spouses. These differences lead to varied behavioral activities and spatial preferences for the older adult, especially in multi-generational households where responsibilities like caring for grandchildren increase overlap with children’s activities. The evident issue of insufficient parent–child space allocation in Longyueju underscores the need for specific improvements. Adhering to the design principles mentioned earlier, combining dynamic children’s spaces with static spaces for the older adult promotes intergenerational sharing behavior, enhancing outdoor activity comfort (74). (2) Varied completion times for residential areas result in distinct age-friendly construction stages. Lianhuabei Village, built in 1994, lacks adequate parking planning, leading to poor road traffic safety and comfort due to a lack of segregation between pedestrians and vehicles. During age-friendly renovations, prioritizing walking comfort for residents, including the older adult, based on the existing road network pattern is recommended. This involves optimizing the road system by categorizing roads into traffic-oriented and life-oriented, reducing external vehicle parking within the residential area through community volunteer management. This improves route continuity, optimizes outdoor activity pathways for the older adult, and ensures traffic safety.

## 5 Conclusion

The assessment of age-friendly communities not only measures the degree of older adult friendliness in the community but also guides planners and designers in age-friendly construction. In situations lacking a systematically constructed and scientifically developed evaluation tool, this study establishes a comprehensive framework for a universal community age-friendly evaluation system. The framework includes the effective evaluation processes, collaborative relationships among multiple stakeholders, hierarchical evaluation content, and diverse evaluation methods. At the practical survey level, using Shenzhen’s affordable housing communities as a case, the study applies the framework to construct an age-friendliness assessment system for public spaces. The system’s effectiveness and applicability are validated, enabling targeted monitoring and evaluation of older adult friendliness. The study also explores common design principles and distinctive strategies for age-friendliness improvement in affordable housing communities, highlighting the impact of built environments, economic levels, policy environments, and other objective factors on age-friendly construction.

However, the limitations of this study pertain to the implementation of the age-friendly evaluation system, wherein the construction of age-friendly communities is regarded as a sustainable, cyclical, and continuously improved process. The evaluation, integral to the closed-loop sequence of “evaluate-understand-plan-implement-evaluate,” is inextricably linked with the other three steps. Consequently, after a period of continuous monitoring and evaluation, the evaluation system may become inapplicable due to alterations in the built environment. To address this, future iterations could employ dynamic indicators and incorporate a feedback loop system, transforming the evaluation system into a responsive tool that ensures continuous optimization in accordance with actual conditions.

## Data availability statement

The original contributions presented in the study are included in the article/supplementary material, further inquiries can be directed to the corresponding author.

## Author contributions

JH: Conceptualization, Data curation, Formal analysis, Investigation, Methodology, Software, Validation, Writing – original draft, Writing – review & editing. HM: Conceptualization, Funding acquisition, Methodology, Resources, Supervision, Validation, Writing – review & editing. MW: Conceptualization, Funding acquisition, Methodology, Supervision, Validation, Writing – review & editing. JL: Methodology, Supervision, Writing – review & editing.
